# How a compensated kidney donation program facilitates the sale of human organs in a regulated market: the implications of Islam on organ donation and sale

**DOI:** 10.1186/s13010-022-00122-4

**Published:** 2022-07-28

**Authors:** Md. Sanwar Siraj

**Affiliations:** Department of Government and Politics, Jahangirnagar University, Savar, Dhaka, Bangladesh

**Keywords:** Kidney donation, Organ transplantation, Organ selling, Remuneration, Bioethics, Islam

## Abstract

**Background:**

Advocates for a regulated system to facilitate kidney donation between unrelated donor-recipient pairs argue that monetary compensation encourages people to donate vital organs that save the lives of patients with end-stage organ failure. Scholars support compensating donors as a form of reciprocity. This study aims to assess the compensation system for the unrelated kidney donation program in the Islamic Republic of Iran, with a particular focus on the implications of Islam on organ donation and organ sales.

**Methods:**

This study reviews secondary documents for philosophical argumentation and ethical analysis of human organ donation and sale for transplantation.

**Results and discussion:**

According to Islamic law, organ donation is an act of *sadaqatul jariyah,* and individuals are permitted to donate organs with the intention of saving lives. The commercialization of humans as organ sellers and buyers is contrary to the Islamic legal maxim *eethaar*, undermining donors of ‘selfless’ or ‘altruistic’ motivations. Such an act should be considered immoral, and the practice should not be introduced into other countries for the sake of protecting human dignity, integrity, solidarity, and respect. I, therefore, argue that Iran’s unrelated kidney donation program not only disregards the position of the Islamic religion with respect to the provision or receipt of monetary benefits for human kidneys for transplantation but that it also misinterprets the Islamic legal proscription of the sale of human organs. I also argue that the implementation of Iran’s unrelated kidney donor transplantation program is unethical and immoral in that potential donors and recipients engage in a bargaining process akin to that which sellers and buyers regularly face in regulated commodity exchange markets. Conversely, I suggest that a modest fixed monetary remuneration as a gift be provided to a donor as a reward for their altruistic organ donation, which is permissible by Islamic scholars. This may remove the need to bargain for increased or decreased values of payment in exchange for the organ, as well as the transactional nature of ‘buyer and seller’, ensuring the philosophy of ‘donor and recipient’ is maintained.

**Conclusions:**

Offering a fixed modest monetary incentive to organ donors would serve to increase organ supply while protecting donors’ health and reducing human suffering without legalizing the human organ trade.

## Background

The program of kidney donation and transplantation between unrelated donor-recipient pairs in the Islamic Republic of Iran is different from that of many other countries in the world [1–4]. Similar to biomedical practices in many other countries, organs have generally been obtained from deceased, living-related, and unrelated transplant donors in Iran [1, 3, 5–8]. Despite the fact that in Iran deceased donors (DDs) or their families do not receive any monetary benefits, every living donor (e.g., related and unrelated) receives a fixed monetary stipend from the government for donating organs [9]. They also receive a year’s worth of medical insurance, transplantation, costs, and medicines at subsidized prices from the government [1, 4, 8, 9]. Aside from the fixed financial compensation from the Iranian government, each living unrelated donor also receives extra monetary compensation directly from the recipient [9]. This compensation is the result of a direct negotiation between the potential donor and recipient on an agreed amount for the exchange of a kidney [1, 4, 9, 10].

This study is based on and adapted from the fourth section of the dissertation project [11]. This article reviews a selection of medical-legal, bioethical, anthropological, sociological, philosophical, and Islamic classical literature and evaluates the stipulations of Iran’s compensation system for unrelated kidney donation for transplantation. It also examines the implications of Islam on organ donation, organ sales, and monetary compensation for human organ transplantation in the light of Iran’s unrelated kidney donation program.

I start with a brief summary of the provisions of the law and practice on the compensated and regulated system for unrelated kidney donation for the transplantation program in Iran. I thereafter proceed to a review of the pertinent Islamic literature, demonstrate the disregard for Islamic law of Iran’s program, and propose a fixed modest monetary incentive for organ donation as a suitable ethical alternative.

### A system for the regulated compensation of unrelated kidney donors in Iran

The first Iranian kidney transplant was successfully performed at Shiraz University in 1967 [8, 12]. Until 1988 saw the advent of a program for kidney donation from unrelated individuals, the number of patients with dialysis steadily increased and hemodialysis was the best option for patients with end-stage renal failure in Iran [8]. From 1967 to 1985, only 112 kidneys were transplanted in the country [4]. The number of patients with hemodialysis increased significantly during the Iranian revolution of 1979, which resulted in the freezing of Iranian assets in international accounts and the eight-year Iran-Iraq war from September 1980 until August 1988 [4]. Economic sanctions by donor agencies and foreign countries resulted in a lack of government funds for the dialysis program, leading to a shortage of equipment. During this time, as kidney transplant facilities were very limited in the country, the Ministry of Health and Medical Education (MOHME) began permitting patients with dialysis to undergo transplantation in overseas countries [4]. Under this procedure, patients with dialysis needed to apply with the required documents and identify living donors to whom they were related (termed living related donors; LRDs) in order to be accepted by the Transplant Centers overseas. Those willing to undertake such transplantation operations could apply for government funding [4]. This system created problems for dialysis patients, as a large number of patients had to wait for transplantation to be granted by the MOHME [8]. In spite of the long waiting list for transplant patients, from 1980 to 1985, using government funds, more than 400 dialysis patients traveled to many European countries and the United States for renal transplantation [8, 13]. This significant increase in the demand for kidneys ultimately led the Iranian Government to establish a legal, compensated and regulated system for renal donor organ transplantation between unrelated donor-recipient pairs [5].

Given that expenditure on kidney donation for transplantation abroad was expensive, the high cost of transplantation certainly increased the waiting list of transplant patients [8]. As a result, a large number of patients with end-stage renal failure endured a long waiting time for their hemodialysis and transplantation. This grim reality officially encouraged Iranian policymakers and health experts to set up transplant centers across the country [8]. Under this initiative, two kidney transplant teams were organized between 1985 and 1987, and only 274 kidney transplants from LRDs were performed by these teams [3]. Another reason for the initiation of a compensated and regulated system for unrelated kidney donation was that deceased and LRDs were the only sources of transplantation organs [8]. This was because a large number of patients with renal failure needed transplantation, but had no potential LRDs for transplantation or, in some cases, their potential related kidney donors refused to donate to their relatives [8, 14, 15].

Despite the program of unrelated renal donor organ transplantation starting in 1988 [4, 8], the initiative to provide financial compensation to living unrelated donors (LUDs) began in 1997 [1, 4]. A non-related kidney donation program was considered a safe and cost-effective procedure with acceptable risk to donors and a ready solution to a scarcity of organs and long waiting times for transplant patients [16]. The reason for introducing compensation for LUDs was the recognition by Iranian society that receiving kidneys from LUDs would increase the donation rate [1]. For example, the increase in the number of transplant centers from two in 1985 to 23 in 2001 resulted in clearing the country’s waiting list for kidney transplants by 1999 [4, 5]. The increase in the availability of human organs, especially kidneys, in Iran was because transplantation was permitted following the confirmed brain death of the donor (with a requirement for close cooperation between Muslim jurists and medical experts) and because of the establishment of a national coordinating body for organ transplantation and the introduction of a compensated unrelated kidney donation program [17].

How does the unrelated kidney transplant program actually work in Iran? In the early phase, once a patient has been identified, physicians search for medically appropriate LRDs for transplantation. In doing so, physicians advise patients to identify potential LRDs within their families [8]. Physicians encourage relatives to donate kidneys to their patients because they have a longer graft survival rate [8]. If the recipient does not have LRDs or a potential LRD is not willing to donate a kidney, the recipient is referred to the Dialysis and Transplant Patients Association (DATPA) to identify organs, particularly kidneys, from DDs, waiting in the queue up to a maximum of 6 months [18]. If no suitable DD is identified in this time, the DATPA searches for an appropriate LUD [18]. Despite families of DDs not receiving any financial stipend from the government, LUDs receive a fixed stipend from the Iranian government, as well as medical insurance, transplant and hospital charges and medicines at reduced prices [4, 9]. LRDs do not usually accept money from the recipient families because their motives are to support a loved one [9].

The provisions on the practice of unrelated kidney donation are divided into three phases [4]. In the first phase, LUDs aged 18–35 who wish to donate kidneys are referred to the DATPA for free registration for both donors and recipients [4]. In the second phase, informed consent is sought from both donors and their immediate relatives to obtain a national identification card from the DATPA [4]. The DATPA then formally introduces a potential donor to the recipient. At this stage, the consent form or “letter of agreement” is duly signed by a witness (e.g., parent or spouse) ([1], 271). The consent form states that the LUD will receive a fixed amount of compensation (10 million Iranian rials) and one-year of post-operative medical insurance and hospital charges from the government or the charity after transplantation [1, 4]. However, for the final evaluation, all donors and recipients are referred to nephrologists for further evaluation, cross-match, and angiography [4]. A complete medical check-up for potential LUDs is performed as donors may have serious transmissible diseases such as hepatitis B and HIV infection [1]. However, tissue matching between organ donors and recipients is performed prior to transplantation. Since the transplant program protects the safety of donors by establishing a screening program and health check-up protocols, it also “rules out the possibility that donors with poor organs may try to cover up medical problems to participate in the program” ([1], 271). If a potential donor has some negative health consequences, he or she will be excluded for transplantation. If a donor is female, either related or unrelated, physicians will pay close attention to any indirect family pressure, resistance or coercion. If females do not wish to donate willingly, physicians will not wish to make a transplant, explaining the cause of medical inadequacy for donors [4].

The third phase directly involves a “negotiation” between the potential LUD and recipient, where the LUD receives extra financial compensation from the recipients for their donation ([4], 629). It usually takes place on the foundation or university premises, where a reserved space is provided for their negotiation [4]. This means that donors meet with recipients prior to donation to confirm payments from recipients under the DATPA control [19]. The DATPA has no record of the agreed amount for the exchange of kidneys and has no role in the negotiation process [4]. In addition, the DATPA only maintains certain controls or formalities over the issue by introducing another potential donor to the recipients in cases where the LUD requires an unusual amount of monetary compensation [4]. Such avaricious donors may be removed from the potential donor lists [4]. The additional reward from recipients is not “regulated” as recipients and donors meet directly where there is no chance of being abused by brokers ([4], 629). It is a government-controlled organ transplantation system where no surgical team or brokers are permitted to participate in a monetary transaction and no intermediary receives any payment. All financial transactions are settled directly between the LUD and the recipient.

After transplantation, donors submit transplant documents, including a hospital certificate certifying that transplantation has been performed, to the designated charity to receive a gift (10 million Iranian rials) and a year of medical insurance [4, 20]. Transplants are performed in university hospitals and are paid for by insurance companies and the MOHME [4]. If a financially poor recipient is unable to pay extra compensation to the LUD, DATPA then seeks assistance from charities to pay the extra compensation to the LUD. By the end of 2006, a total of 21,359 kidneys had been transplanted across Iran, of which 15.2% came from LRDs, 5.2% from DDs and 79.6% from LUDs [17].

## Methods

This study reviews secondary documents for philosophical argumentation and ethical analysis of human organ donation and sale for transplantation.

## Results

### An assessment of the compensated kidney donation program in Iran

Iranian scholars who support an unrelated kidney donor organ transplantation program often argue that monetary compensation or reward for kidney donation to LUDs is permissible as it saves the lives of many vulnerable patients [4, 8]. Further, it is associated with acceptable donor risks, reduces the scarcity of transplantable organs through a ‘safe’ and ‘cost-effective’ procedure, and decreases the death rate for patients with end-stage organ failure while on the waiting list [16]. The Iranian government’s financial compensation scheme for LUDs is an effort to address many issues plaguing Iranian society, including increasing unemployment and poverty, poor dialysis patient outcomes, and the black market in organs [3]. The LUD compensation scheme was created in response to a perceived shortage of organs available for transplantation, and it was intended to promote organ donation through financial incentives [9]. Bagheri expresses concerns about the direct payment system between donors and recipients for the exchange of kidneys but he supports the offer of additional financial compensation packages to LUDs, arguing that the LUDs should not be deprived of their “rightful claim to be compensated” because everyone who participates in the procurement of organs, except the donor, should be responsible for the recognition of the sacrifices of organ donors ([1], 270). Bagheri asserts that there is no conflict between an altruistic act of organ donation and a logical compensation for one’s organs, time and financial loss ([1], 279). In addition, a justification in favor of a compensated and regulated unrelated kidney donor organ transplantation program is offered by Abdallah Daar:

“[I] f the buying and selling of organs is as unstoppable as it appears to be, then leaving it totally unregulated causes more harm than good, which is arguably unethical especially as it encourages only the rich to benefit. Regulating the practice will very likely minimize harm by opening it to scrutiny, enforcing compliance with standards to protect donors, recipients, and society, removing rapacious middleman, and enabling the poor to receive transplants on an equal footing with the rich. What could be a more obvious step if we want to improve the situation?” ([21], 601).

Nourbala et al., therefore, suggest that other countries apply Iran’s experience to enhance their kidney donation for transplantation programs because it has good health outcomes and low costs compared to programs in other countries ([22], 929).

What are the rewards for altruistic donations in Iran? As mentioned earlier, an LUD currently receives 10 million Iranian rials from government or charities, a year of health insurance, expensive medicines at subsidized prices, and additional compensation from the recipient. For additional compensation, if potential recipients are unable to pay LUDs, there are charities that can help them.

The fixed amount of compensation (10 million Iranian rials) could be referred to as an ‘altruistic gift’ as donors and recipients are not involved in the negotiation of kidney exchange. Medical facilities such as subsidized medicines and 1-year’s free health insurance are necessary for donors to recover in good health. However, what seems most controversial is the additional compensation paid directly to the LUD by the recipient. The transplant surgeon Anne Griffin considers it to be an unregulated system because it involves inadequate oversight monitoring of human organs ([23], 502). Although Iran’s scholars consider all monetary compensation to be rewarding altruistic donation, unrestricted bargaining situations between donors and recipients for organ exchange seems more like organ selling and purchasing, as such practice is very similar to hidden markets in human organs elsewhere in the world [24–29]. Despite Iran’s unrelated kidney donation scheme saving many lives of vulnerable patients, at the same time it provides an opportunity to engage in bargaining for human organs and could simply be called organ sale. Given that potential donors and recipients individually meet and engage in negotiations, it should be considered a completely free bargaining in organs like free trade, which is unacceptable, ethically impermissible and morally flawed. Iran is the only country in the world to have legalized the human kidney trade with LUDs [1, 5, 30].

As has happened before, with many patients currently on the waiting list for organ transplantation in Iran, the huge demand for kidneys could create an unwelcome bargaining situation between unrelated donors and recipients. Despite Iran’s biomedical legislation suggesting that LUDs who demand unreasonable compensation will be excluded from the donor list, barring these LUDs from the list is perhaps coincidental as several transplant patients are still in the long queue for transplantation. For example, a single-center study report shows that the average waiting time for renal transplantation is 386.22 days [5]. Perhaps very few potential donors have been barred from the list as rich patients are in need and healthy organ donors, particularly the poor, are looking for a quick fix to solve their financial problems. Malekshahi et al. argue that people in lower socio-economic groups may decide to sell their kidneys to mitigate their financial problems: LUDs are, therefore, mostly motivated by economic reasons [5]. As Harmon & Delmonico explain, “there is no oversight of these transactions in the Iranian system, nor could such oversight be assured or verified in any other regulated market” ([31], 1146). In Iran, not only are poor donors intentionally chosen on the basis of economic class, but Iran’s policy makers, physicians, researchers, and bioethicists must realize that permitting LUDs an unlimited bargaining position will encourage and coerce poor donors to sell their kidneys to rich recipients.

Overall, the debate raises the question of whether this transaction should be permitted by biomedical policy and what amount of monetary compensation or reward should be set for living altruistic donations. Although some scholars are in favor of selling human organs to markets for the sole purpose of saving human life [24, 32–35], many Islamic institutions, as well as the vast majority of Muslim scholars and physicians still do not support this ([14], 178 [36];, 39–40). Unlimited bargaining conditions make the transplantation process a disgrace, as a study shows that only poor individuals sell their kidneys to rich patients [9] because poverty makes poor Iranians vulnerable to exploitation [37]. Iran’s poor donors are forced to become organ vendors as they are mostly “helpless,” “jobless,” “indebted” and “largely destitute” ([38], 625). However, it is hopeful and encouraging that the Iranian government has recently diverted funds from the unrelated kidney transplant program to DD transplantation ([38], 626), and the now 14% of transplants from deceased donors indicates that some progress has been made in deceased kidney transplantation in Iran [5]. Nevertheless, kidneys have mostly been “purchased” from LUDs for transplantation ([5], 4). For example, a recently published single centre study shows that approximately 51.8% of the kidneys received were purchased and 48.2% were donated altruistically ([5], 3). Nejatisafa and others think that the Iranian model of organ transplantation should be reassessed and overhauled before offering it as a successful model to the transplant community in the rest of the world ([39], 940).

## Discussion

### Implications of Islam on organ donation and transplantation

Islam is a holistic religion that directs every aspect of human life [40]. According to the Islamic tradition, humans are the greatest creations of God on Earth (Quran 95:4). However, God has made humans susceptible to disease and ailments [41]. The Prophet Muhammad (Peace Be Upon Him, PBUH) has said that “Allah did not send down a disease without having sent down its cure” ([14], 162). The Quran and Prophetic sayings encourage Muslims to actively seek remedies for their ailments [41, 42]. Islam encourages Muslims to seek new methods of treatment and to apply them if they have proved successful [43]. Organ donation and transplantation is a new invention of modern science and technology that has emerged as a lifesaving medical treatment for patients with end-stage organ failure [11]. As all aspects of human life including science and technology in general are part of Islamic teaching, modern science and the medical invention of organ transplantation, in particular, is, therefore, a part of Islamic culture. So, the issue of organ donation and transplantation should be interpreted appropriately in light of the Islamic traditions and its legal jurisprudences. As organ donation and transplantation is a new medical procedure only available since the twentieth century, the issue is not explicitly mentioned in the verses of the primary sources of the Islamic classics such as the Quran (the literal word of The Almighty Allah as dictated to Prophet Muhammad [PBUH] by the archangel Gabriel) and the Sunnah (sayings, actions, works and tacit assents of the Prophet Muhammad [PBUH] and his companions) directly [36, 44–49]. As the Quran and Sunnah were written in the seventh and ninth centuries, it is not possible to find direct rulings in the primary sources of Islamic jurisprudence on the permissibility of organ donation and transplantation [36]. Muslim scholars thus prefer to use secondary sources such as the *Ijma*, the general consensus; *Qiyas*, the inference, and analogies; and *Ijtihad*, the exhaustive efforts of Islamic scholars to find the explanation on a particular modern issue in light of the primary sources [14, 47]. If a clear ruling on a particular issue can not be found in the secondary sources, Muslim scholars then prefer to use subsidiary sources such as the *Istihsan*, preferential reasoning; *Urf*, customary conventions; and *Maslahah*, the cannon for public welfare and the common good, to find guidance on the issue derived from the guidelines of the primary sources of Islamic law [14, 47].

Muslim scholars who oppose and support organ donation for transplantation use almost the same verses of the Quran and Sunnah [14, 47, 50, 51], but their opinion about the permissibility of the issue varies with their interpretation of the texts. Geographical and historical differences, cultural and societal diversity, prevailing social customs, and the variety of politico-administrative systems have inevitably led to differing views among Muslim scholars about whether organ donation should be permitted [52]. The variance of opinions between Sunni and Shia communities regarding organ donation further complicates the issue and results in disparities in the laws on organ transplantation between Muslim countries, depending on which sect is in the majority [36]. Sunni Muslims, accounting for between 85 and 90% of total Muslims, and the remaining 10 to 15% Shia both share their juridical guidance of legal orthodoxy as follows: the *Maliki*, the *Shafi*, the *Hanbali*, the *Hanafi*, and the *Jafari*. Muslims who belong to the *Maliki* school live today in sub-Saharan Africa and Egypt; the *Shafi* believers are currently active in the Indian sub-continent, East Africa, Egypt, and Yemen; the *Hanbali* Muslims are dominant in Saudi Arabia; and the *Hanafi* Muslims are widely spread out in the Muslim region and historically prevalent in Turkey, central Asia, Europe, the Middle East, Afghanistan, and the Indian Sub-continent including India, Pakistan, and Bangladesh [14]. Shia Muslims are the majority mostly in Iran, Iraq, Azerbaijan, and Bahrain. While there is a diversity of opinion among Muslims regarding the permissibility of organ transplantation across the world, scholars from the Indo-Pakistani subcontinent who belong to the *Ḥanafī* school tend to hold contingently permissible views and Shia scholars in the Middle East often hold views that the process is permissible as-is [45].

Due to problematic interpretations of Islamic classic literature and fatwas, Muslim adherents often hold varying opinions on the permissibility of organ donation and transplantation. Consequently, Muslim scholars have discussed the ethical and legal consequences of organ transplantation for many years [52]. Since Arabic remains the dominant language for such debates, discussions, and publications, little is known about the implications of Islam on organ transplantation in the wider international bioethics community [52]. Prejudice regarding the permissibility of organ transplantation is widely disseminated, which contributes to confusion. For example, contentious fatwas banning organ donation are often posted on social media, while those advocating for the biomedical practice are not. Even though a fatwa might be more commonly applied by individuals due to its prominence, it does not give it an inherently higher ethical-legal status [53]. These variations have resulted in a diversity of opinions among Muslim scholars about the permissibility of organ donation and transplantation.

Most Muslim scholars, Sunni and Shia, support organ donation and transplantation [47, 51, 54] but their opinions are not universally accepted, as some hold the view that organ procurement contradicts *sharia* Islamic law [36]. By citing the following Quranic verses (2:195; 5:32; 8:27; 17:70), scholars who oppose organ donation base their stance on the Islamic belief that the human body, whether dead or alive, is a sacred thing that should be treated with care, respect, compassion, and utmost attention [55]. It is thus disrespectful to violate the human body in some way to procure organs or body parts [56]. These scholars reject organ donation because it abuses the human body [36]. Muslim scholars also oppose organ transplantation because human life is a divine gift and the human body, including its integral organs, is an *Al-Amanah* or a sort of trusteeship that Muslims receive from The Almighty, not the sole property of humans themselves (Quran 4:58). Muslims should therefore maintain this trusteeship [51]. Humans can not donate any organs which are not their own [47]. Because The Almighty has endowed humans concerning their bodily organs (Quran 90:8), giving consent for someone to obtain organs for transplantation by harming one’s body is a breach of trust with The Almighty, which is not permitted in Islam [49]. Muslim scholars also maintain that altering The Almighty’s creation is not accepted in Islam (Quran 30:30). They hold the view that opening the human body to obtain an organ is an act of aggression against the human body and these types of aggression should not be permitted [41, 49]. Scholars view organ donation as an impermissible activity based on the principle of the sanctity and entrustment of the human body. They use the following Prophetic saying “Breaking the bones of a Muslim when he is dead is like breaking it when he is alive” as justification for their argument as aggression toward the human body to be a crime [41]. They also assert that The Almighty will not accept those who alter His creations by cutting the human body and obtaining organs for transplantation as they have committed a wrongdoing. They warn that wrongdoers who alter The Almighty’s creation will be punished in the a*l-akhirah* or the hereafter [51].

Scholars who oppose donating organs for transplantation are in the minority and form small groups among contemporary Muslim scholars and believers [36, 47, 51]. They narrowly focus their argument as they generally use the literal and strict meaning of the verses of the two primary holy scriptures, the Quran and Sunnah [47]. In contrast, scholars supporting organ transplantation base their views on the spirit of the verses of the Quran and Sunnah. They argue that modern medical science has proved organ transplantation to be a successful method of treatment [11]. They support organ donation, as the purpose of Islamic law is to serve the betterment of human society, so organ transplantation should be endorsed legally as it provides benefits rather than degrades the welfare of humankind [47].

Muslim scholars who endorse organ transplantation cite several Islamic principles to support their argument. The Islamic principle based upon the verse of the Quran (2:173) as “*al*-*darurat tubih al-mahzurat*” or “*necessity renders all prohibited things permissible*” is often cited by Muslim scholars to justify organ donation [14, 36, 48]. That is, while organ donation is prohibited in Islamic law, it is permitted if there is a dire necessity. Religious scholars thus approve organ donation for transplantation to preserve lives even though violating the human body is forbidden in Islam. Organ transplantation is conditionally permissible by Muslim scholars based on the principle that the pressing needs of the living outweigh the benefits of the dead. The group of scholars who base their view on the Islamic principle of ‘dire necessity’ generally cite Quranic verses (2:173; 6:145; 16:89) to support their argument.

Altruism or *eethaar* is highly praised in Islam [36]. Saving the life of a human holds great importance in the Quran and Sunnah. Scholars favoring organ transplantation repeatedly refer to the verse of the Quran (5:32) that supports human altruism based on “… whoever saves a life, it will be as if they saved all of humanity”. The Quran encourages helping others and saving a human life [36, 44, 49]. Islam values saving human lives and prioritizing the needs of the living over the dead. The altruism framework thus permits humans to use their bodies correctly and encourages individuals to donate their organs as gifts [40]. The two Prophetic sayings that Islamic scholars apply to organ donation and transplantation are s*adaqatul jariyah* (an ongoing charity) and *kullu-ma’aroofin-sadaqah* (all good deeds are a charity) [36]. That is, donating organs to someone in dire need of a life-sustaining transplant is a long-term act of charity. Since organ donation is s*adaqatul jariyah*, a donor will be rewarded as narrated by Prophet Muhammad (PBUH), “when a person dies, all their deeds end except three: a continuing charity, beneficial knowledge, and a child who prays for parents”. The interpretation of the Prophetic saying is that organ donation is one of the most continuing acts of charity that a person performs because the benefits of this type of charity can be reaped both during one’s lifetime and long after one has passed away.

The following Quranic verse is also cited by Muslim scholars to validate organ donation: “cooperate with one another in righteousness and piety, and do not cooperate in sin and transgression. Have fear of Allah. Allah is stern in punishment” (5:2). The interpretation of this verse is that Islam encourages Muslims to be careful and cooperative to their communities in ‘righteousness’ and ‘piety’, not in ‘sin’ and ‘transgression’. Muslim scholars thus endorse organ donation as it sustains or prolongs human life [47]. As saving the life of a vulnerable patient is a good act, Muslim scholars affirm that such cooperation is surely a good deed [47], and The Almighty will reward Muslims in this life for their good deeds, as well as in the a*l-akhirah* or the afterlife (Quran16:97).

There is also widespread agreement among Muslim scholars and Islamic organizations on the permissibility of living and deceased donor organ transplantation issued in similar rulings, including those from the United Kingdom Muslim Law Council, the Islamic Jurisprudence Assembly Council in Saudi Arabia, the Islamic Code of Medical Ethics, the Islamic Religious Council of Singapore, the Medical Ethics Committee of the Islamic Medical Association of North America, and the Islamic Organization of Medical Sciences [36, 41]. The International Seminar on Organ Donation and Transplantation organized by the Indonesian Council of Ulama together with the Federation of the Islamic Medical Association (FIMA) and the Indonesian Forum for Islamic Medical Studies in Jakarta on 30 July 1996 determined that the human body and its internal organs belong to The Almighty (Quran 2:195) and these divine assets were created for the welfare and benefits of humankind (Quran 2:29), so humans can use organs for the welfare and benefit of the community [14, 51]. It is lawful to obtain organs from a living person that can regenerate such as the bone marrow, skin, a part of the liver, or any non-regenerative organs without which a donor may still survive (e.g., one kidney or a portion of a lung) but organs (e.g., heart, pancreas) which are essential for survival can not be donated. Further, donors must be adequately informed about the potential outcome of transplant procedures [41]. This condition upholds the spirit that Islam preserves by saving human life as stated in the Quran (5:32) ([48], 3273), donating an organ that is not one of a pair to a recipient is risky for donors because it may harm their health. So humans can donate organs that can save the lives of patients that would not put donors in grave danger. Scholars suggest that organs can be removed from a body of brain-dead donors for transplantation if donors or their families give consent and physicians can declare a person dead medically with good understanding [41, 49].

### Provisions of Islamic law concerning organ sales

While most Islamic scholars endorse organ donation, there is significant disagreement concerning the permissibility of organ sales ([57], 4–5 [36];, 39–40). Most scholars oppose accepting any form of compensation for organs on the basis that the human body and its integral organs are not commodities that can be sold [48]. These scholars base their view on the Islamic principle that, as previously stated, the human body and its integral organs are the sole property of Almighty Allah, and humans are merely its caretaker or steward [58, 59].

Some scholars believe that both the donation and sale of organs are permissible in Islam only when there is no other option available to save a life of a patient. They argue that if the existence of necessity makes the prohibited act permissible (i.e., conditionally permitting someone to donate organs for transplantation), then there is no good reason why such Islamic principle ought not to be used for organ sale. If the life of a patient depended on purchasing an organ, would it not be permitted out of the principle of necessity? A moderate Egyptian Sunni Muslim scholar, Mufti Muhammad Sayed Tantawi, answers the question by arguing that both the donation and sale of human organs are permissible only when there is no other option to save the life of a patient as the existence of dire necessity would make a prohibited act permissible [60]. Tantawi also argues that an organ can be purchased and transplanted into another human body in dire necessity as the removal of greater harm and preserving a human life are the highest priorities in Islamic scriptures such as in the Quran and Sunnah [60]. If the Islamic principle of ‘dire necessity’ can be applied to organ donation, what Tantawi claims is that it can also be applied to organ selling. That is, a person in dire need of an organ should be permitted to buy it for transplantation. Most Sunni scholars oppose payment for organs, while Shia jurisprudence has ruled in favor of payment if the organ is required to save a life [41].

### Islamic interpretation opposing organ selling

Muslim scholars who support organ donation for transplantation are primarily against organ sales ([47], 734). The Indonesian Council of Ulama in the final Resolution of the International Seminar on Organ Transplantation and Health Care Management from Islamic Perspective with the Indonesian Forum for Islamic Medical Studies (IFIMS), the Indonesian Council of Ulama and the Federation of the Islamic Medical Association refers to the following three propositions: 1) the advocates of the school of thought defined as “*milku–al-raqabah”* (right over the whole body including internal organs), regard humans to be the owners of their whole body and its internal organs; humans, therefore, can sell their organs or give their organs to others; 2) the advocates of the school of thought defined as “*milku-al-manfa’ah”* (mere right over the organs), consider that humans have mere rights over their organs; so humans have rights to use their organs and this in turn stipulates the right to lend, but not sell, them; and 3) according to the school supporting “*milku- al-intifa”* (a sort of trustee over the organs), humans have the right to use their organs only for their own benefit. Humans do not have the right to lend or sell their organs to other people ([14], 162–163). The *Ulama* (a body of Muslim scholars) who joined this seminar unanimously agree and support the third school of thought *Milku-al-intifa* [14]. It follows that humans have the right to use their organs, but as the human body belongs to The Almighty, they cannot lend or sell their organs to anyone else. The Resolution concludes that humans have the right to use their organs only for themselves but not to lend or sell their organs to anyone.

The Islamic Fiqa Academy in Jeddah in the final announcement of a conference on organ transplantation in March 2009, prohibited humans from selling their bodily organs [57]. The reason, as the Academy announced, is that “the human body is sanctified by The Almighty who had forbidden turning it into an item for commercial sale, purchase, or exchange. Humans must be a reliable guard of his body” ([57], 5). Many other Islamic organizations such as the United Arab Draft Law on Human Organ Transplants (article 7, adopted by the Council of Ministries of Arab countries, Khartoum, 1987) prescribe that the selling of human organs or financial remuneration is always prohibited and that doctors must not participate in or facilitate such an organ transplant if informed of such negotiations [14]. The reason is that the resulting physical health of recipients is not good after such transplantations [14]. An example from Kuwait can be used in this regard. Many organ failure patients went abroad, bought organs, and underwent transplantations between 1986 and 1990. After returning home, many patients had to go to the medical center at Kuwait University due to negative and serious health consequences, including tuberculosis, rejections, infections, contagious diseases, and various forms of hepatitis; four of the transplant recipients were HIV positive and two of these died of AIDS [14].

The Muslim scholar Abdulaziz Sachedina states that the human body is not a commodity that can be turned into a commercial deal or another advantage ([59], 187). The human body cannot be used as a means of negotiation in other than exceptional and unavoidable circumstances. Sachedina argues that organ transplantation is only conditionally permitted based on Islamic jurisprudence as it ensures greater benefits than harms ([59], 185). Muslim scholars warn that The Almighty has imposed restrictions on how humans use their bodies and their integral organs. As organs are the parts of basic human dignity, they can be donated, but engaging in bargaining for their sale is immoral because human life deserves dignity and integrity, making organs worthy of care and protection. Supporting this position, Dr. Amin Muhammad Salam Al-Batush, a Wahhabi scholar, asks the question “is the human body the property of its owner?” ([57], 5). He gives the answer “there is no law, nature, or logic which could permit the sale of human body parts, since The Almighty sanctified and separated humans from other things” ([57], 5). He argues that “saleable goods are those which are detached from the human being, not which are connected to him” ([57], 5). This implies that human organs are an integral part of the human body and should not be considered commodities or resources for sale or that which can be used to solve a financial crisis or to fulfill a basic need [45].

Among Muslim scholars who oppose organ sale, Sherine F. Hamdy states that Egyptian jurists favor organ donation in certain circumstances when there is no alternative for saving a human life without transplantation. She reiterates that there should be no harm that afflicts living donors and no commercial transactions involving organs [61]. She argues that the necessity (*Darura*) over the welfare of human life (*Maslahah*) makes organ donation conditionally permissible without exchanging financial benefits [61]. A prominent Egyptian Islamic scholar, Sheikh Zaki Badawi, issued a ruling that states “human organs should be donated, but not sold. It is prohibited to receive a price for an organ” ([62], 156). By citing Quranic verse (90:13), scholars determined that the donor of an organ should receive no financial benefit, effectively ruling out any organ payment system as a viable policy [36]. This means that human organs should not be sold in markets as commodities, but can be donated based on *eethaar* or selflessness, altruism.

Muslim nephrologist, Yassin Ibrahim M. El-Shahat, argues that organ donation is an act of altruism, charity, and benevolence through which the lives of humans are saved ([48], 3273). He persuasively argues that human organs are not commodities and should only be donated for the spirit of love and cooperation with one’s fellow humans ([48], 3273). He argues that viewing one’s organ as a commodity that can be sold in markets is directly an affront to human solidarity and dignity and is strictly forbidden in Islam ([48], 3273). As selling anything involves bargaining, such practice may force donors and recipients to engage in competition for higher and lower prices. Islam condemns such practices because everything, including human organs, belongs to The Almighty (Quran 2:195; 4:29) and, as such, human organs should not be considered commodities. If humans are forcefully placed in markets, they would count as mere objects ([63], 207). As a result, human organs will necessarily become commodities with price tags, so they would no longer be priceless, worthy, and valuable [64]. Organ selling would also undermine donors’ altruistic motivations to donate organs [63]. Selling and buying human organs in a market would disregard the act of organ donation as a gift and undermine its charitable obligations to save a human life [63]. Bioethicist Dariusch Atighetchi argues that monetary bargaining for human organs reduces the altruistic inclination in people and in the relatives of the patient to donate their organs ([14], 180).

### Why does Islam prohibit organ selling?

The practice of organ selling and buying is prohibited in Islam on the basis of several ethical considerations: a) “human dignity” ([48], 3273); b) “exploitation of the poor” ([1], 274 [26];, 345 [47];, 731 [65];, 772); c) “sacredness of human life” [66], 222 [67];, 1326); and d) “respect for humans” ([57], 5–6). This section explains why the sale of human organs for transplantation is not permitted in Islam.

#### Human dignity

Scholars see the practice of organ selling as contrary to “human dignity” ([68], 362). Organ selling is strictly prohibited in Islam because it erodes human dignity, placing humans on the market for sale as commodities ([69], 223). While people openly and freely sell and buy many things that sustain human life without having to deal with any moral complaints, the human body and its integral organs are not ordinary products that can be exchanged. They are not produced like caps, hats, t-shirts, and boots, and should not be sold for the need of anyone else. If a human body and its integral organs are considered as objects for exchange it would imply that humans, in general, can be treated as mundane entities, which would undermine human dignity. Islam requires that we recognize the human body and its integral organs as a gift from The Almighty on Earth ([47], 726), so donors may only take risks with their bodies to save the lives of their loved ones inspired by altruism and solidarity.

Human organs (e.g., kidneys, liver, pancreas, eyes, etc.) are integral parts of the human body and these are necessary for human life [70–72]. Without these, there is no possibility of sustaining a natural life [73]. Advocates of organ sales treat the human body as a “collection of spare parts” ([32], 10), but all organs in humans are integral insofar as they are necessary for normal existence ([73], 143–144). If we lose one of our kidneys or eyes, we can survive, but not the same as before. While the brain is a higher organ than the kidney or liver, a human body is created with two kidneys, and both kidneys are important for complete bodily function. Living life with two kidneys is not equivalent to living with only one. As such, a kidney is an indispensable part of the human body, and the removal of a kidney may cause serious injury or even death. Physicians suggest that people who have a single kidney should be careful with the rest of their life and protect it from injury and that it is best to avoid contact sports such as football, cricket, boxing, hockey, soccer, martial arts, or wrestling [74]. From October 1999 to December 2008, 14 living kidney donor deaths (0.03%) were reported to the Organ Procurement and Transplantation Network or identified in the Social Security Death Master File among 51,153 donors within 30 days of donation and 39 donors (0.08%) had died by 12 months after donation [75]. As such, all organs are integral insofar as they are necessary for good health and natural existence. An example from Sherine F. Hamdy also supports this argument:

Why did God give us two kidneys? Just as there are two eyes for the function of complete sight and two ears to hear all 360 degrees around us, so are the two kidneys essential to filter toxins throughout the body. With two kidneys, there is extra energy for us to absorb exposure to external or internal insults to the body. When we take out one of the kidneys, there is inflation in the remaining kidney, proving that the function rises. Sometimes a kidney donor will need occasional dialysis because both kidneys are necessary. Like the fibers in our muscles - most of the time, they compensate for one another. Each takes turns working; they don’t all work at the same time. It is like the alternating keys on the piano. God created humans in this way. Each kidney has one million nephrons, and only 10 percent work at a time, taking turns. Why? Why did God give us so much function? The function of the kidneys is to make the toxins in the urine stay under a particular level in the blood … … Because the kidneys carry out such a huge important task, there has to be two of them. And there’s a relationship between them; they compensate for one another. They call this counterbalance. Like the liver, you take a piece of it, a lobe, and transplant it into someone else. And this small lobe will grow to the size of a normal liver, and then it stops. Why doesn’t it keep on growing? How does it know which size to grow? The cells have a memory; these are divine signs that no one understands exactly. Now when you take a piece of this liver from the donor, there are great risks involved. He could die, the donor. When we go on a car journey, we carry a spare tire in the car. And life is an eternal journey, [so] we need the reserves that God gave us. This is why organ transplantation from living donors creates harm to the donors. And we have a legal-ethical principle in Islam: la darar wa la dirar [no harm can be inflicted or tolerated]. And that prevention of harm takes precedence over taking a benefit ([73], 143).

So, all integral organs are essential for healthy survival. Without doing serious harm, we should recognize that Islam only permits organ donation for transplantation in cases of extreme necessity to save a life ([48], 3273), so donors may only take the risk to assist and save the lives of loved ones and sick patients inspired by the spirit of altruism and solidarity. The sole purpose of selling organs, however, is to profit financially, which may put poor people in particular on the market as interchangeable commodities.

#### Exploitation of the poor

Muslim theologist, Ghulam Haider Aasi, considers that organ selling directly exploits the human body and the poorest and weekest members of society, thus selling parts of it for monetary benefit is forbidden in Islam ([47], 731). Aasi opposes organ sale because monetary incentives may be used to coerce poor and vulnerable people in any society into selling their body parts or organs to wealthy patients.

Moniruzzaman ([76], 69), Moazam, Zaman, and Jafarey ([77], 29), and Gill and Sade ([78], 29) see the relation between the sellers and buyers as exploitative, unlike donors who donate altruistically. As most organ sellers are in extreme poverty, they cannot make objective decisions due to their vulnerable position ([79], 146). In contrast, despite being in need, recipients are always the winner in purchasing organs from poor people because buyers are comparatively rich, educated, and well informed, and they can easily convince and coerce poor sellers ([76], 75 [80];, 17 [81];, 53). In addition, organ recipients will usually try to buy organs by paying as little as possible to the vendor. Organ selling is actually not a level playing field as the poor vendor is always coerced by monetary interest and has limited decision-making capacity and bargaining power in terms of fully comprehending the health consequences of organ donation ([82], 1267). Iran is a glaring example of where poor people sell their kidneys to rich recipients [83]. Thus, it is clearly suggested by the World Health Assembly to its member countries that each country has “a responsibility to protect the vulnerable and poor from being exploited as a source of organs for the rich” ([84], 1414).

While legalizing a market in human organs would increase the capacity to supply human organs for transplantation, it would also place humans in markets as objects and exploit poor people because the poor remain vulnerable in bargaining situations. For example, LUDs likely sell their kidneys in Iran where a vast majority of kidneys (76%) are currently procured from impoverished donors who often use their money to pay off debts [18]. An empirical study of a randomly selected sample of donors showed that most (84%) LUDs in Iran were from poor areas, 16% belonged to the middle class, and none of them were from wealthy sectors. Of those who received kidneys, 50.4% were poor, 36.2% belonged to the middle class, and 13.4% were wealthy [85]. Kidney recipients may belong to the poor socio-economic class in Iran as it is possible to seek help from charities to pay LUDs for donating organs [19], but the vast majority of LUDs are from socio-economically impoverished sectors of society, and poor vendors are directly involved with recipients in the asymmetrical negotiation process for kidneys.

#### Sacredness of human life

Organ selling is “inhumane” and “unacceptable” ([86], 961) as rich recipients wish to purchase healthy organs for securing and sustaining life. Wealthy buyers typically want to purchase healthy kidneys from young sellers. For instance, a cross-sectional study in Iran found that LUDs were younger compared with LRDs ([87], 3210). If selling organs is legally permitted, the poor may be encouraged to sell their organs whatever the medical risk. The principle of Islamic Jurisprudence, however, is to protect human benefit that outweighs the risk. Julian Koplin argues that Iranian kidney vendors endure a range of potential harms as they desperately want to sell their organs to get out of poverty ([88], 8). Moniruzzaman also suggests that Bangladeshi poor people sell their organs and take on high risks of suffering ([26], 171). Organ selling thus erodes the sacredness of human life as only the rich recipient derives benefits because only they can afford to buy organs, violating the principle of justice [89].

Theologian and bioethicist Alastair Campbell states that seeing the human body as an indefinite object which is interchangeable with other objects and commensurable with monetary values is morally wrong ([90], 17). Mario Morelli reinforces the moral dictum of Emanuel Kant and sees human beings as moral agents and ends in themselves, not as merely a means for others to gain an end ([91], 318). What Morelli reveals is that if one donates a kidney for the purpose of beneficence, one does not use oneself as a mere means. Giving up a bodily organ for other reasons, such as financial gain, however, violates human dignity. Treating the human body as a commodity with a monetary value for the exchangeable object is always treating someone as a mere means to an end. This secular viewpoint is consistent with Islamic principles, which consider the human body and its integral organs as sacred things [47, 49]. While advocates of the market in human organs argue that those who are rich have legal and moral rights to buy anything that they wish [32, 34, 35, 92], payment for organs is necessarily degrading and incompatible with basic human values such as social justice, equality and the spirit of solidarity and altruism. Organ selling or the establishment of a regulated market in human organs is very likely to cause harms that outweigh the benefits to sacred human life [83, 88].

#### Respect for humans

Saruhan opines that each human action is evaluated based on intentions ([93], 84). This means that intention is the essential component for the moral evaluation of an action. Intention is the motivating force that engages humans in their actions. Considering this view, I argue that saving a human life by donating organs altruistically is different from selling organs. Despite the risk of surgery and the potential for serious complications in either donating or selling organs**,** both violate the sanctity of and respect for human life. However, the bodily violation is diminished in Islam if it is overwhelmed by the act of improving others’ welfare and good ([94], 334).

Philosopher and bioethicist Mark J. Cherry sees no intrinsic difference between the practice of organ donation and sale, as the main purpose of both is to preserve human lives, eliminating greater harm ([32], 152), but an ethical question arises as to whether these two things are equivalent. For example, Mark Cherry argues that “if it is altruistic for a parent to give a kidney to a child to save his life, it can similarly be altruistic for a parent to sell a kidney to pay for the lifesaving operation” ([32], 152). Despite there being no difference between the intention of a father who wishes to save the life of a child and either donating organs or paying monetary benefits by selling organs for a child’s life-saving surgery, I consider that the nature of such actions is different. Despite the similar intention of the father, the actions are different. Saving the life of a child by donating organs is morally permissible as it would not place the father on the market as an exchangeable commodity. On the other hand, saving the life of a child by selling an organ and paying for a life-saving surgery is a morally wrong action because the human body is not a mere thing, but a blessing and trusteeship from The Almighty Allah (Quran 4:58). The Prophet Muhammad (PBUH) prohibited selling what one does not have ownership of (e.g., Tirmidhi 1232). We should thus consider that everyone is reasonably entitled to act differently based upon their particular intention. Islam emphasizes that a person who has planned to donate altruistically will not be blamed for his action in practice as he has good intentions (i.e., to save human lives) [95, 96]. Such an action is perceived as an altruistic expression and moral commitment to save human life [97]. On the other hand, organ selling may place the human body in a market as an everyday product, ignoring and undermining the essential gift as one has the intention of receiving monetary benefits rather than saving the lives of others. Islam thus permits organ donation for transplantation altruistically [97–99], while selling organs characterizes the human body and its organs as exchangeable assets [100] to be used to support the benefits and interests of the buyer [19, 76]. The respect for humans is disregarded when the human body and its integral organ are intrinsically considered as exchangeable objects and products.

#### If blood, why not organ sale?

Many might argue that if selling blood is legally and ethically permissible in critical circumstances, why not sell human organs. Their argument, the comparison between selling human blood and organs, however, is misleading and inappropriate. We must recognize the fact that human blood and human organs are not the same. As previously mentioned, necessity often makes unlawful things permissible [48, 52, 101]; when there is no alternative way of saving a life, human blood can be sold to save that life. Blood selling is unlawful [14, 102], except when it is the only option to save human life [14], and otherwise should not be permitted. This interpretation is based on the verse of the Quran that states that ‘O Prophet’, “I do not find in what has been revealed to me anything forbidden to eat except carrion, running blood, swine—which is impure—or a sinful offering in the name of any other than Allah. But if someone is compelled by necessity—neither driven by desire nor exceeding immediate need—then surely your Lord is All-Forgiving, Most Merciful” (Quran 6:145). The consensus of scholars is that blood selling is permissible when it is carried out to save a human life, but they argue that this illegal practice should never be permitted. If humans are permitted to sell their blood, it would turn an altruistic act into an illegal practice. On the other hand, human blood and organs are different. Human blood is a replaceable tissue, unlike the internal organs of the human body, and blood donation does not cause as serious a degree of harm to the blood donor’s health as is caused by organ donation. As such, trade in organs should not be permitted as the practice has negative health consequences for donors and seriously dishonours them through the forfeiture of “irreplaceable body parts” ([94], 338), while blood selling does not harm the donor in the same way. Moreover, being a blood donor may have some positive benefits; one example is cited in the *American Journal of Epidemiology* that finds blood donors are 33 and 88% less likely to suffer from cardiovascular disease and heart attack, respectively ([103], 448)*.*

### Legal provisions in Islam addressing gift rewarding

The World Health Organization (WHO) resolution “*preventing the purchase and sale of human organs”* asserts that the purchase and sale of human organs for transplantation is exploitative and incompatible with human dignity [104]. This resolution contends that prohibition of organ selling is necessary so as “to prevent the exploitation of human distress, particularly in children and other vulnerable groups, and to further the recognition of the ethical principles which condemn the buying and selling of organs for purposes of transplantation” [104]. Therefore, the WHO urges member countries to adopt appropriate measures to enact policies and regulations forbidding commercial transactions involving human organs [104]. Despite the WHO prohibition on organ selling and monetary transactions involving human organs, in 1987 a well-known Indian Physician, Dr. C. T. Patel, first introduced the term “gift with reward”. Patel argued that “kidney donation is a good act. It is the gift of life. The financial incentive to promote such an act is moral and justified” ([105], 22). Patel was actually justifying the practice of receiving compensation for LUD kidney transplants. Thus, the term ‘rewarding gift’ entered into the professional debate in bioethics literature.

What are Muslim scholars’ views on rewarding the gift of organ donation for transplantation? Dariusch Atighetchi sees that there is a difficulty in distinguishing between the sale of human organs and the practice of rewarding the gift received for donations ([14], 178). The reason is that, in both cases, recipients and donors desperately engage in bargaining for organs that makes the issue most critical and debated. The International Islamic Fiqa Academy issued a ruling that, despite organ selling being strictly prohibited, considered monetary gifts to altruistic donors as a debatable issue ([106], 2044). While the Jeddah Council of the Academy of Islamic Law (Resolution no. 26 on organ transplants, 6–11 February 1988) bans organ selling completely, it emphasizes that recipients could consider bearing the expenses or paying compensation to donors as a sign of appreciation that is necessary for altruistic donors’ survival [14]. Sahin Aksoy’s rewarding the gift of altruistic organ donors is a very persuasive argument. Aksoy claims that despite Islam prohibiting organ sale, the giving of a reward for altruistic organ donation can be permitted as Islam does permit exceptions ([107], 468). Aksoy recognizes that human organs are not mere property that can be donated freely and should not even be considered a legitimate part of trade or a way of earning or generating income, but that does not mean that any financial transaction associated with organ donations should be considered forbidden ([107], 468). As Islam permits exceptions, offering financial benefits to donors for altruistic organs does not infringe the sprit of the Islamic principles as it is a natural way of life ([107], 468). As our nature limits human choices and freedom of actions from doing anything that one wishes, one cannot do whatever one wishes with one’s own body. As such, Natour & Fishman are against giving any compensation, price or gifts for altruistic organ donations as Muslim donors may come to expect a heavy reward for their altruism ([57], 6–7). But I support Aksoy ([107], 469) as he views compensation in the form of a limited degree of reward (e.g., half the blood money such as 5000 Kuwaiti Dinars) for altruistic organ donations as a way to encourage donations. He sees financial reward as a mechanism to motivate donors as it can be considered an example of the robust realism of the Islamic way. Aksoy also suggests that some amount of monetary compensation can be offered to donors under state supervision if it encourages people to donate organs and promotes public welfare ([107], 472) According to Aksoy, a limited reward would not coerce altruistic donors to donate organs against their altruism as the reward is limited and fixed. Rather it may motivate donors altruistically to donate organs for transplantation where a regulation should be set under state authority. The government can incentivize charitable donations by providing medals, free medical treatments, or even a set amount of money for the well-being of a donor’s health [108].

Limited and fixed financial benefits as rewards for receiving organs is expressing an appreciation and honor to altruistic donors. Egyptian Islamic scholar Sheikh Yusuf Andullah al-Qaradawi, who opposes organ selling because human organs are not “merchandisable things” to be “bargained over,” believes altruistic donors may receive a gift or a gift of honor (*ikramiyya*) from unrelated beneficiaries ([57], 5). What he expresses is that bargaining for human organs is prohibited in Islam but offering rewards to altruistic donors as gifts is permissible. The main objective of offering a modest benefit for organs is to give assurances to altruistic donors that the donation does not involve bargaining, but is voluntary, and human life is protected, not exploited or coerced. The permanent committee of a supreme Islamic Judicial Authority in Saudi Arabia issued a ruling that “there is nothing wrong with accepting it (an amount of money as a gift), without you (the recipient) longing for that, and you can respond in kind if you are able to with an appropriate gift, or you can supplicate for him, because the Prophet Muhammad (PBUH) is reported to have said that “Whoever does you a favor, respond in kind, and if you can’t find the means of doing so, then keep praying for him until you think that you have responded in kind” [109]. This Prophetic verse can be interpreted as offering gifts intrinsically is a moral appreciation by recipients that shows friendship and honour to altruistic donors and is legally and ethically permissible in Islam.

As discussed previously, organ donation is a voluntary, altruistic and charitable act [110–114]. Many might argue that altruistic donors might not expect anything in return for donations. I contend that if recipients wish to give some benefits for receiving organs freely, why ought we not to consider it for altruistic donors. Altruistic donors may also receive such benefits. Offering modest benefits as rewarding gifts for altruistic donors should not be considered a morally offensive practice because it would not involve any bargaining and it may not coerce altruistic donors into selling their organs to anyone beyond their altruism. These benefits should be considered as a moral and thankful appreciation by the recipients that do not place altruistic donors or their organs in markets as commodities.

When a Muslim carries out a good deed, he or she reserves his or her intention for The Almighty and does not expect any reward in return, as Muslim believers should continue to strive for rewards in the hereafter while rewards are not necessarily seen in the world (see Quran 4:40). Despite Islam not supporting the expectation of receiving a reward in return for good deeds, I contend that offering some benefits intrinsically to altruistic organ donors is not a payment in return for a generous act but consistent with Islamic principles which define the act as being committed without expectations. Insofar as human organs are precious, valuable and sacred things, these are characterized as gifts and should always be donated in the spirit of solidarity and altruism [69, 115, 116].

In a subsequent address to the Transplantation Society, Pope John Paul II also stated that any practice that attempts to commercialize human organs or treats them as exchangeable products must be deemed ethically impermissible [30, 117]. While the Christian Church has long opposed payment for organs donated by living persons, ‘entitlement compensation’ for human organs is permitted because it retains the act of donation as essentially altruistic ([36], 40). In addition, from a Jewish religious moral standpoint, giving a reasonable compensation to donors for their act of ‘self-endangerment’ in saving a life is a good act ([118], 423).

Despite Bagheri’s view having been criticised as he supports giving unlimited financial compensation to altruistic LUDs in Iran, his claim is partially substantiated as he sees depriving the donor of receiving gifts or thankful wishes for their generous donations in many societies worldwide as driving forward the acceptability of a regulated market in human organs ([1], 272). For example, the absence of financial compensation for altruistic donors’ post-operative care and well-being has created a black market in many countries of the world, including India, Pakistan, The Philippines, Iran, Iraq, Egypt, Bangladesh and many others [76, 77, 119]**.** My normative argument is that if we may not willingly offer something for receiving organs, will sufficient altruistic donors continue to donate organs willingly? Glasson et al. [120], Delmonico et al. [121], Grazi & Wolowelsky [122], Friedlaender [24] and Novelli et al. [123] have explored various types of incentives for altruistic organ donation, such as providing healthcare expenses, tax relief, educational grants for their children, or pension/early retirement benefits, as well as funeral costs in the case of the deceased donor, to alleviate the current shortage of organs.

If altruism is the motivation for organ donation, and as organ donation is considered a charitable gift to the recipient, why should it not be permitted to give any gifts to the donors in return for their altruistic donation? One viewpoint proposed by Delmonico et al. [112] is that the symbolism of gift giving is critical as it is a core social value in many societies. However, while these writers support the notion of offering gifts in return for altruistic organ donations (akin to the Red-Cross giving t-shirts, food, drinks, etc. in their blood donation programs), they are against offering any gift with a financial value. In another publication, Delmonico and his colleagues ([121], 1187) oppose payment for human organs but propose mitigating the burdens on organ donors such as the costs of travel and accommodation for medical examinations and organ retrieval surgery, and loss of wages and expenses during the period of organ removal surgery and recovery, which is described as a neutral act. It is possible to take a different view of the symbolism of gift giving. Although offering a reward (in the form of a modest amount of money) for receiving organs has a monetary value, it should still be considered as a gift. It is a benefit offered in gratitude. Any gift can have monetary value, just as t-shirts, food, or drinks do. The crucial point is that gifts are not to be used as commodities, with their value to be bargained for in an open market. In the case of organ donation, the monetary value of the reward must not be so much as to attract the poor or vulnerable to donate purely in order to receive the reward. As long as these conditions are met, then the offering of a limited and reasonably modest gift in exchange for receiving organs is symbolically appropriate, as long as the organ recipients benefit from the donations. Delmonico et al. ([112], 2004) also believe that the fundamental trust of society including life and liberty should not have any monetary price. They argue that its value is “disregarded when a poor person feels compelled to risk death for the sole purpose of obtaining monetary payment for a body part” ([112], 2004). I contend that this does not mean that the recipient should never offer any monetary reward to the donor. If such reward is permitted, then it should be offered to the donor as a limited amount, without negotiation, and primarily to express the recipient’s appreciation of or gratitude to the donor for their assistance and acknowledgement of the inconvenience endured. This should not lead to organ trade or the attribution of a price tag to any organ.

As donors donate organs altruistically, recipients may also offer something else in reward to the potential altruistic donors such as the Iranian government or charities offering a fixed amount of 10 million Iranian rials. It is not just that those who receive an organ for transplantation will have their life saved or improved; those who donate altruistically will not receive any benefits from transplantation. I consider that offering a fixed modest benefit to altruistic donors would be a fair acknowledgement of the organ donation for transplantation. Donors and recipients each benefit from such an altruistic donation and the subsequently expressed thanks. This symbolic gift or financial benefit may vary from country to country and region to region with variations in Gross National Product, Gross Domestic Product, and costs of medical and social care, and daily living expenses. A number of scholars have proposed a fixed amount of financial compensation for organ donors [30, 124, 125], but these come with a larger price tag. I believe that a higher financial incentive for exchanging organs will compel altruistic donors to donate their organs for reasons other than altruism.

A limited benefit may secure donors’ safety and thus reduce the potential harms that donors may incur through donating organs for transplantation. We must recognize that the fixed amount of money, is not for the organs, but this benefit is rightly set at a reasonably generous level to show a moral appreciation and religious obligation to the donors. Otherwise, if such benefit is not offered, donors may not always be willing to donate because they may feel worried about their health. Denying such benefits to altruistic donors might equally be considered to be denying patients the right to live.

Medical anthropologist, Monir Moniruzzaman, argues that rewards are highly controversial as they “promote the concept that organs are not bought, rather, donors receive a reward for their gifted organs” ([26], 328). He cites two references in favor of his argument: Lesley A. Sharp notes that “reward gifting is an oxymoronic euphemism that downplays the contradictions inherent in attempts to blend altruistic and market principles; rewarding gifting and direct payment occupy different points on the same continuum” ([26], 328); and Robert M. Veatch argues that “rewarding gifting is a blatant corruption of the language as it signifies that the transfer of money is not a ‘reward’, but a payment” ([26], 328). What I understand is that both Lesley Sharp and Robert Veatch see reward gifting as a (direct) payment. We should recognize that payment (direct) does not compel donors to donate against their altruism as long as the payment does not attract donors. What I mean is that, despite the reward being direct, if it is moderate, it will not force people to go against their altruism. Furthermore, I contend that if the reward is fixed, there is no chance for donors or recipients to engage in bargaining. Removing the ability to bargain for increased or decreased payment in exchange for the organ removes the transactional nature of “buyer and seller” and ensures the philosophy of “donor and recipient”.

Finally, we ought not to consider this rewarding gift as an exchange of human organs for transplantation. Despite the WHO’s guiding principles and the Istanbul Declaration both opposing material gains or incentives for organ donations globally [126], we should consider that offering a limited, bearable, and fixed rewarding gift for altruistic donation is realistic and pragmatic. First, as healthcare and social welfare coverage is not present in many countries worldwide, altruistic donors may face negative health consequences after their transplantations. As donors need to receive follow-up care immediately after 1 month, 6 months, 1 year and annually thereafter, such fixed benefits will help to assure the full recovery of altruistic donors. After transplantation, donors need costly medication, clinical follow-up care and proper education and counselling on their physical and psychological health and wellbeing. Second, if altruistic donors face negative health consequences, these benefits can properly be used to lessen their physical and psychological suffering. Third, donors need to incur the ancillary costs of organ donation such as the travel expenses, lodging and food while travelling for medical examination and surgery, loss of wages, and other expenses during the period of pre-surgical assessment and organ removal surgery. Reimbursement of these costs should not be considered as the price of the organ; rather it should be considered as part of the expense of follow-up care. If such benefits are not provided to altruistic donors, donors may not always donate their organs freely. As the question of financial compensation for receiving organs becomes unavoidable in the current socio-economic climate in many parts of the world, it is logical that altruistic donors may not always wish to donate altruistically without receiving reimbursement where the safety net of health and social care coverage is virtually absent. Despite the Council of Europe’s “Additional Protocol to the Convention on Human Rights and Biomedicine Concerning Transplantation of Organs and Tissues of Human Origin” strongly prohibiting individuals from benefitting financially from donations, this does not prohibit donors from receiving justifiable compensation for their inconvenience to cover post-operative costs and expenses in regard to the loss of income [127]. If we were not to do this, it would denigrate this charitable practice into the immoral and unethical practice of organ selling. Consequently, organs will only be sold to medically suitable patients who have the ability to pay vast quantities of money for the privilege.

## Conclusions

In conclusion, gifts in reward for donating organs are not akin to payments, as long as no bargaining is involved that may exploit a donor’s vulnerability and/or recipient’s corrupt intentions. The reward for organs must be fixed and modest. That it is limited is crucial because if donors could receive unlimited financial benefits from recipients, it may compel poor people into donating their organs without altruism, as currently happens in Iran. A fixed, modest reward would not coerce or exploit the vulnerable and would not compel them to donate organs purely for financial gain because the value of the gift would be absolute. As this gift is fixed (e.g., 10 million Iranian rials) for altruistic donors to express the recipient’s moral and thankful appreciation for receiving organs, such gifts would not encourage donors to sell organs to improve their circumstances in life. Such a gift is not controversial or unethical. As there are several direct and indirect costs associated with organ donation and post-operative care, a pre-defined gift is necessary to ensure the donor’s full recovery and well-being. Despite Islam opposing bargaining between donors and recipients for the exchange of organs, it supports offering a fixed gift for altruistic donation. Permitting such a rewarding gift would be an effective, efficient, and ethical means of obtaining organs for transplantation that will increase the supply of human organs for transplantation, protect donors from harm, and reduce human suffering without legalizing organ trade. It may be time for the government of Iran to revise its public policy and practice in biomedicine to prevent the poor from selling their organs in a regulated market.

## Data Availability

Not applicable.

## Abbreviations

DATPA: Dialysis and Transplant Patients Association
DD: Deceased Donor
LRD: Living Related Donor
LUD: Living Unrelated Donor
MOHME: Ministry of Health and Medical Education
PBUH: Peace Be Upon Him
WHO: World Health Organization
